# Seismic velocity structure of Unzen Volcano, Japan, and relationship to the magma ascent route during eruptions in 1990–1995

**DOI:** 10.1038/s41598-021-00481-6

**Published:** 2021-11-17

**Authors:** Kanta Miyano, Koki Aizawa, Takeshi Matsushima, Azusa Shito, Hiroshi Shimizu

**Affiliations:** 1 Graduate School of Science, Kyushu University, Fukuoka, 819-0395 Japan; 2 Institute of Seismology and Volcanology, Faculty of Science, Kyushu University, Shin’yama, Shimabara, Nagasaki 855-0843 Japan; 3 Faculty of Biosphere‐Geosphere Science, Okayama University of Science, Okayama, 700-0005 Japan

**Keywords:** Solid Earth sciences, Volcanology

## Abstract

Subsurface structures may control the migration of magma beneath a volcano. We used high-resolution seismic tomography to image a low- P-wave velocity (Vp) zone beneath Unzen Volcano, Japan, at depths of 3–16 km beneath sea level. The top of this low-Vp zone is located beneath Mt. Fugendake of Unzen volcano, which emitted 0.21 km^3^ of dacitic magma as lava domes and pyroclastic flows during eruptions in 1990–1995. Based on hypocenter migrations prior to the 1990–1995 eruptions and modeled pressure source locations for recorded crustal deformation, we conclude that the magma for the 1990–1995 eruptions migrated obliquely upward along the top of the low-Vp zone. As tectonic earthquakes occurred above the deeper part of the low-Vp zone, the deep low-Vp zone is interpreted to be a high-temperature region (> 400 °C) overlying the brittle–ductile transition. By further considering Vs and Vp/Vs structures, we suggest that the deeper part of the low-Vp zone constitutes a highly crystalized magma-mush reservoir, and the shallower part a volatile-rich zone.

## Introduction

**Figure 1:**
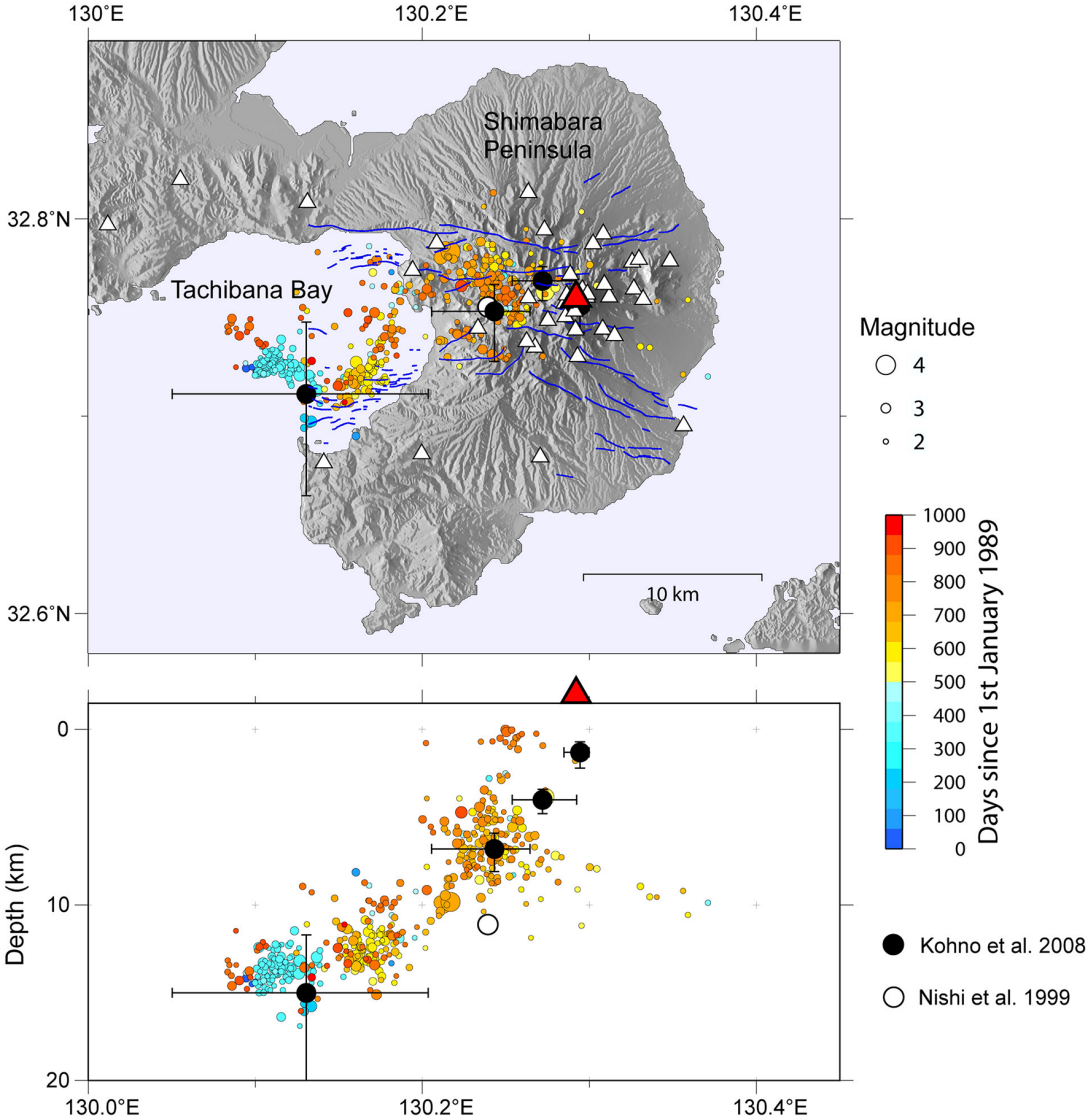
Hypocenter distribution for the 1000 days since 1 January 1989, taken from Umakoshi et al. (2001). The first eruption occurred on 17 November 1990. White triangles indicate seismic stations used in this study. Note additional seismic stations were used in this study that lie outside the region shown above (see Fig. 2a). Red triangle and blue lines indicate Mt. Fugendake and active faults (Tsutsumi, 2015), respectively. Solid circles and lines show the pressure sources and 1σ error bars determined by joint leveling and GPS observations (Kohno et al., 2008). White circle indicates the pressure source determined by GPS observations (Nishi et al., 1999).

Unzen Volcano is located in the central part of Shimabara Peninsula, Kyushu Island, Japan. The volcano is famous for the growth of a new dacitic lava dome, followed by its repeated collapse and the resultant generation of pyroclastic flows, during eruptions in 1990–1995. The total volume of magma erupted during this period is estimated to be 0.21 km^3^ dense-rock-equivalent^1^. Unzen Volcano is situated in an E–W-trending tectonic graben that contains many ~ E–W-trending normal faults^2,3^ (Fig. 1). Development of nested normal faults during the growth of Unzen Volcano has resulted in periodic subsidence of the main edifice and a thick accumulation of volcanic products down to ~ 1 km beneath sea level throughout the 500 ka history of the volcano. The volcanism of Unzen is divided into Older Unzen (500–200 ka) and Younger Unzen (100 ka to present), with total erupted volumes of 120 and 8 km^3^, respectively^3^.

Subsurface magma movement related to the 1990–1995 eruptions has been inferred from seismic and geodetic measurements. The hypocenters of tectonic earthquakes gradually shifted from a depth of ~ 15 km, beneath Tachibana Bay, to shallower depths beneath Mt. Fugendake^4^, and this has been interpreted as magma migrating upward to the east at an angle of ~ 45° from the horizontal (Fig. 1). This hypothesis is supported by the results of a GPS study^5^ and a study that combined GPS and leveling data^6^, which indicate pressure sources to the west of the volcano (Fig. 1). This eastward and upward magma route was first suggested by Ohta^7^, on the basis of geochemical analysis of hot spring waters. The results of these previous studies suggest that the primary magma reservoir of the 1990–1995 eruption is located west of Unzen Volcano, possibly beneath Tachibana Bay (Fig. 1). On the other hand, isotope ratio analysis shows that ^3^He/^4^He values (7.0 Ra, where Ra denotes atmospheric ^3^He/^4^He = 1.4 × 10^–6^) east of Shimabara Peninsula (Shimabara Spa) are four times higher than those west of the peninsula (Unzen and Obama Spa)^8^, which suggests that the contribution of magmatic gas is strongest towards the east. The ^3^He/^4^He data are enigmatic and might indicate another magma reservoir east of Shimabara Peninsula.

Estimation of seismic velocity structure using the travel time of natural earthquakes can resolve features down to the depths of the middle to lower crust, and has been employed to image magma reservoirs beneath volcanoes worldwide^9–21^. Two previous studies have used seismic velocity data to image the subsurface of Unzen Volcano. Ohmi and Lees^22^ used the travel times of crustal earthquakes recorded prior to the 1990–1995 eruptions to image a low-velocity zone southeast of Fugendake at depths below 10 km, interpreted as a primary magma reservoir. However, at that time only four seismic stations were in operation on Shimabara Peninsula. Zhao, et al.^23^ analyzed crustal and deep earthquakes associated with subduction of the Philippine Sea Plate beneath Kyushu Island. They identified a large cone-shaped low-velocity zone beneath Unzen Volcano and interpreted it as a high-temperature region containing melts or partial melts. Of note, neither study identified low-velocity zones west of the volcano (beneath Tachibana Bay), which is seemingly inconsistent with the observed hypocenter migration and pressure sources inferred for the 1990–1995 eruptions. It is possible that the spatial resolution of the previous studies was not sufficient to image a potential magma reservoir, given the small number of seismic stations and earthquakes used in their analyses.

In the present study, we estimate the high-resolution velocity structure beneath Unzen volcano by using the travel times recorded by a dense network of modern seismometers. We use these data to discuss the possible locations of magma reservoirs and their relationship to the ascent route of the magma that fed the 1990–1995 eruptions.

## Data and method

We estimate the seismic velocity structure in 3D using the double-difference (DD) tomography method of Zhang and Thurber^24^. The absolute arrival times and relative arrival times are weighted according to their estimated accuracies. Application of the DD tomography method enables us to jointly determine hypocenter locations and the 3D velocity structure using absolute and relative event locations. The pseudobending ray-tracing algorithm^25^ is employed to identify rays and calculate travel times between events and stations.

**Figure 2:**
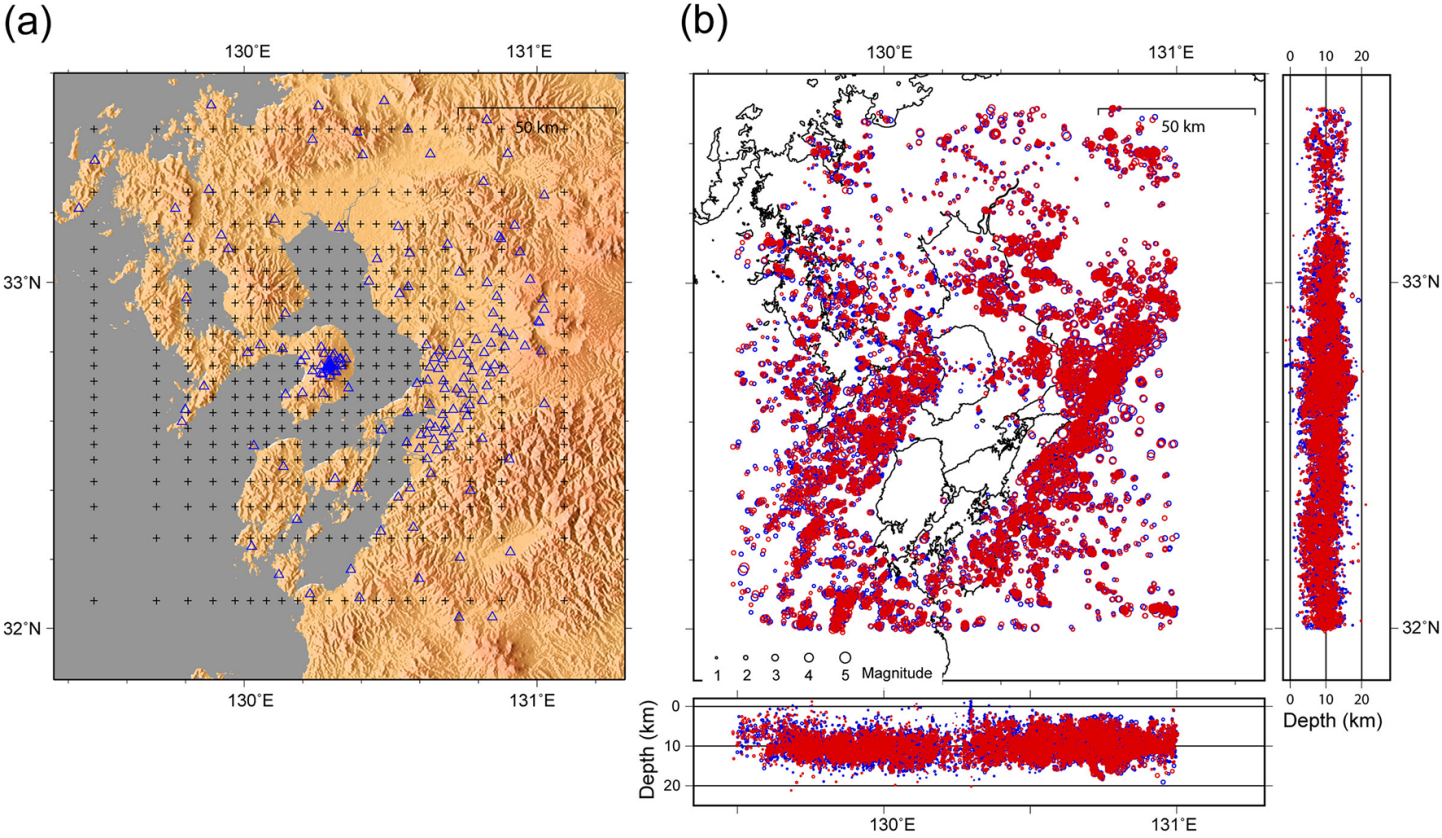
Maps showing the locations of seismic stations and hypocenters. **(a)** Seismic stations (blue open triangles) and grids (crosses) used in the seismic tomography. The grid is centered on Mt. Fugendake (32.76°N, 130.29°E). **(b)** Initial (blue) and relocated (red) earthquake hypocenters. The change in hypocenter locations is shown as histograms in the east–west, north–south, and vertical directions (Fig. S4).

The array of 179 seismic stations used in this study is shown in Figs. 1 and 2a. The stations are operated by Kyushu University, NIED (High-Sensitivity Seismic Network, Hinet), and the Japan Meteorological Agency (JMA). The sampling rates are 100–200 Hz. We used the P- and S-wave arrival times of earthquakes that occurred between January 2002 and March 2020 to construct the seismic velocity structure beneath Unzen Volcano to a depth of 16 km. The hypocenters of crustal earthquakes around Unzen Volcano are not equally distributed in space; e.g., Fig. 2b shows an aseismic zone south of Shimabara Peninsula, possibly related to the presence of strong plutonic rock^26^. The aseismic zone reduces the overall spatial resolution of the structure owing to the reduction of raypaths, but we later confirm that the estimated velocity structure is sufficiently well-resolved for the purposes of this study.

Arrival times were picked manually at Kyushu University. We select events with a minimum of 10 picks. We did not use data for which the picking error of the arrival time exceeded 0.2 s for P-waves or 0.4 s for S-waves. To ensure an equal distribution of the earthquakes in space, we divided the study area into 0.01° × 0.01° × 1 km blocks and selected between 1 and 10 of the largest events in each block. In total, our selection criteria yielded 7614 earthquakes with 224,262 P-wave arrivals and 168,839 S-wave arrivals. The P- and S-wave travel times with respect to the hypocenter distance are shown in Fig. S1. In the inversion, we set the weighting factor to 1.0, 0.5, and 0.25 for the absolute travel time of P-waves according to their picking errors (± 0.05, ± 0.10, and ± 0.20 s, respectively). The S-wave weighting factor was set to 0.8, 0.4, and 0.2 for the absolute travel time according to their picking errors (± 0.10, ± 0.20, and ± 0.40 s, respectively). The DD approach also uses travel time differential data^27^ to reduce relative location errors on hypocenters and better constrain the velocity structure. We used two steps in the travel time difference inversion. First, travel time differences between earthquake pairs within 8 km of each other were used, yielding 923,089 arrivals for P-wave data and 612,313 arrivals for S-wave data. Second, this distance was reduced from 8 to 3 km, yielding 739,210 arrivals for P-wave data and 494,562 arrivals for S-wave data. We also conducted a single-step inversion from earthquake pairs within 3 km of each other, reaffirming the results of the two-step inversion (Fig. S2).

In the model space, we represent the velocity structure on a regular set of 3D grid nodes, between which the velocity values are interpolated using trilinear interpolation. Figure 2a shows the node locations used in the study. The origin (X, Y) of the horizontal coordinate system is centered at Fugendake (32.76°N, 130.29°E). The horizontal grid spacing is ~ 5 km within the area of interest. In the vertical direction, grid points are located at depths of –500, –1.0, 1.0, 3.5, 6.0, 8.5, 11.0, 13.5, 16.0, 20.0, 25.0, and 500 km. As an initial model for the inversion, we use a 1D Vp structure determined from the average crustal value for Kyushu Island^28^. We use 1.73 as an initial Vp/Vs value, which is similar to the average velocity structure of Kyushu Island^28,29^.

**Figure 3:**
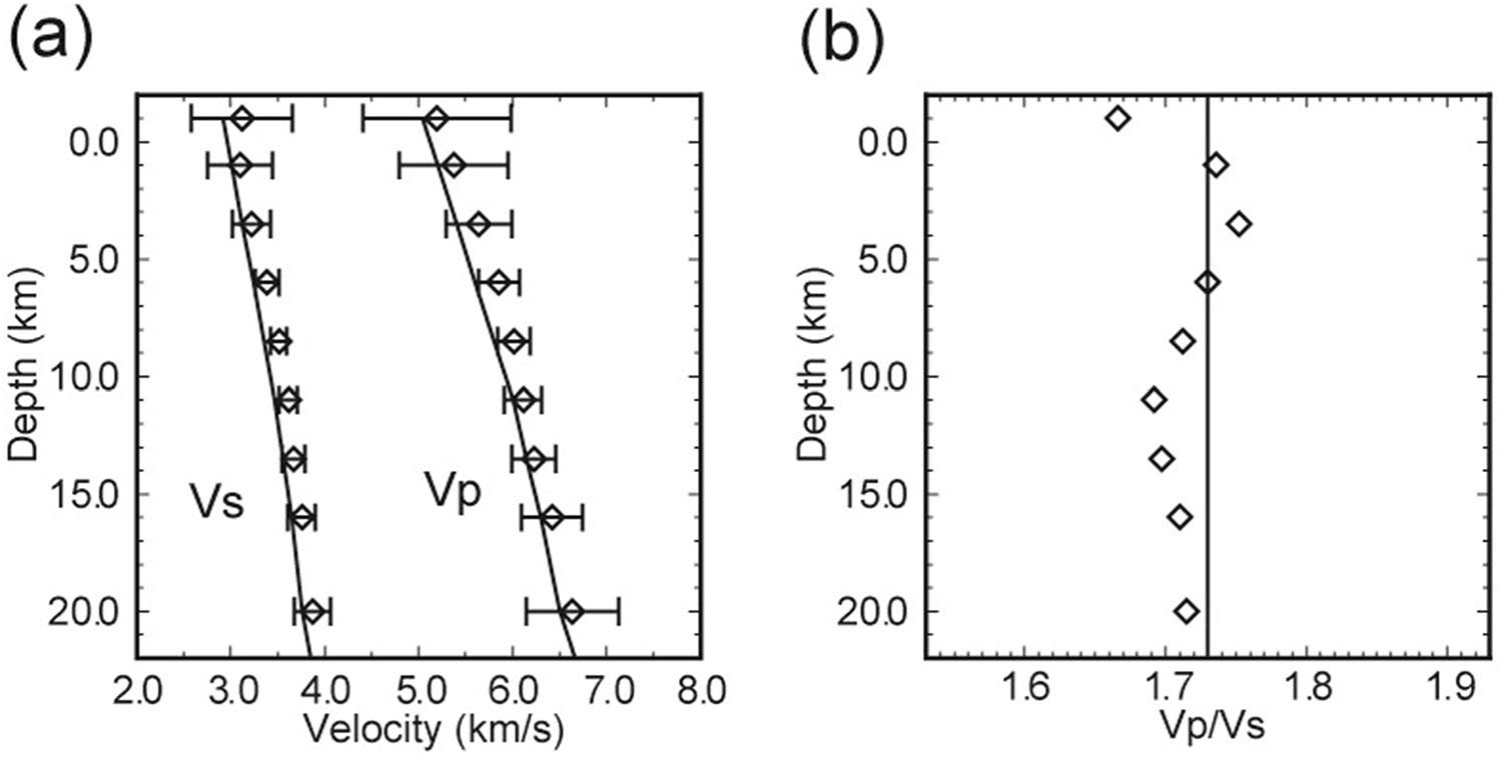
**(a)** Initial (lines) and final (diamonds) one-dimensional seismic velocity models. Final 1D velocities were calculated from a weighted average using the derivative weight sum (DWS). The standard deviations of the resultant model are plotted as horizontal bars. **(b)** Initial (line) and final (diamonds) Vp/Vs ratios.

The inversion process is carried out iteratively, and we obtain a decrease in root mean square travel time residuals from 0.26 to 0.07 s after 16 iterations. The P- and S-wave residual distributions from the initial 1D structures and those from the final 3D structures are shown in Fig. S3. Histograms showing the change between the initial and relocated hypocenters are provided in Fig. S4. Figure 3 shows depth variations in the mean velocity, calculated from a weighted average using the derivative weight sum (DWS) of each grid point^29^ and the Vp/Vs ratio of our 3D model. The DWS measures the ray density in the neighborhood of each node^30^. The variation in estimated velocity is similar to that in the initial model, supporting the validity of the initial 1D approximation. To check the robustness of the inversion, we also conducted three inversions by changing the initial 1D structure and Vp/Vs value. We used a 1D structure from Kyushu University, one from JMA, and one that used 1.55 as an initial Vp/Vs value. The obtained results are shown in the supplementary material (Fig. S5-S7). No significant difference is observed in the region with high DWS, so we conclude that the result is robust when the initial structure in the inversion is changed.

### Resolution

**Figure 4:**
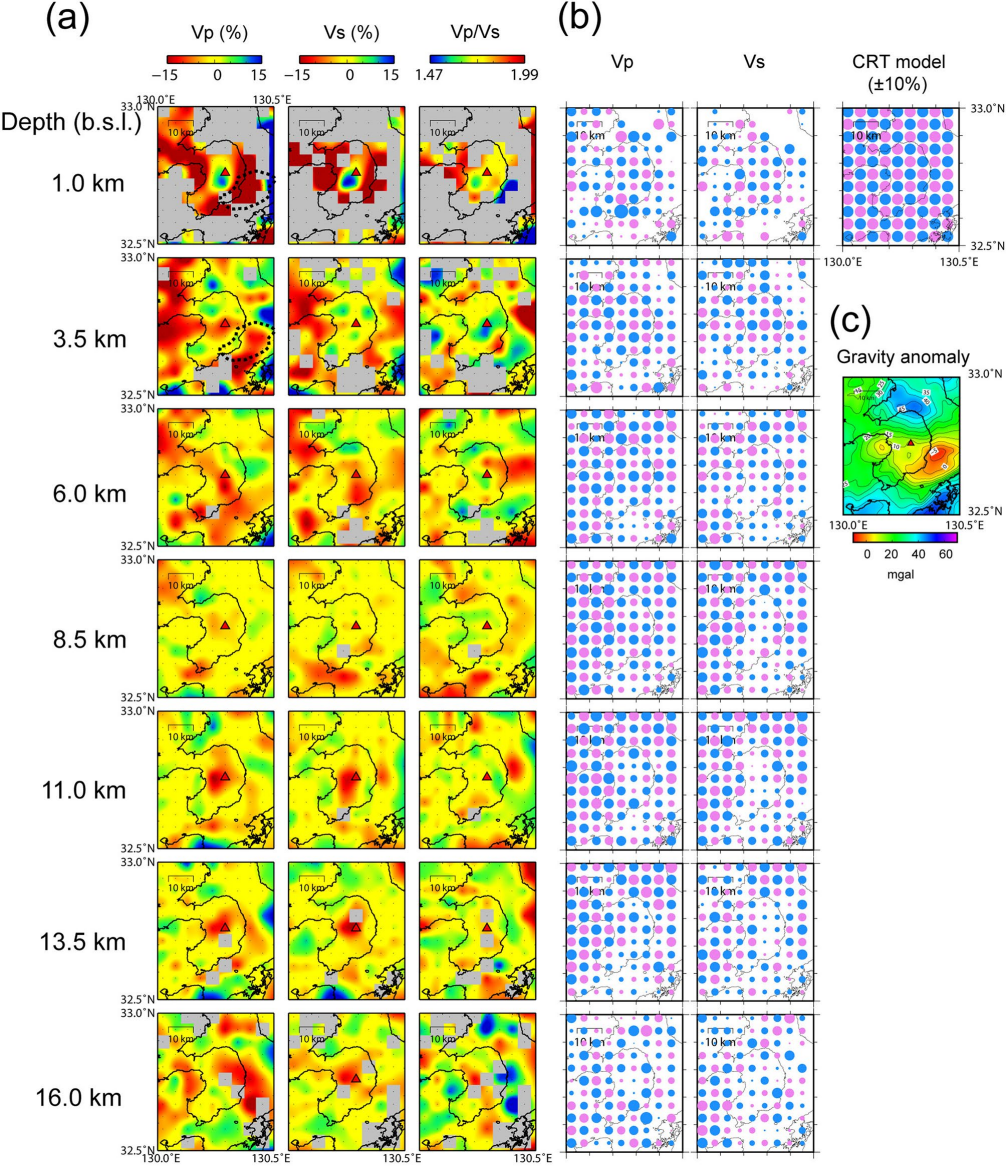
Maps showing the velocity structure and gravity anomaly. **(a)** Horizontal slices of P-wave, S-wave, and Vp/Vs perturbations at various depths (beneath sea level, b.s.l.). The images were created by interpolation of model parameters solved at the grid points shown in Fig. 2a. Perturbations were calculated as the deviation from the DWS weighted averaged 1D structure (Fig. 3). Areas with resolvability values of < 0.65 are masked (gray). The low gravity zone shown in **(c)** is marked by a black dashed line at Vp structure 1 and 3.5 km b.s.l. **(b)** Result of a checkerboard resolution test (CRT). The area of each circle is proportional to the recovery rate from the initial checkerboard pattern (± 10%). **(c)** Bouguer gravity anomaly assuming a terrain density of 2.63 g/cm^3^. The contour interval is 5 mgal. The low-gravity zones are well correlated with the low-Vp zone at 3 and 6 km b.s.l. Other notations are the same as in Figs. 1 and 3. Gravity data for the gravity basemaps are used under copyright from the Geospatial Information Authority of Japan (GSI); the map is licensed under the Creative Commons Attribution 2.1 Japan License. The Bouguer anomaly map was compiled using a database from AIST (Geological Survey of Japan AIST 2013).

**Figure 5:**
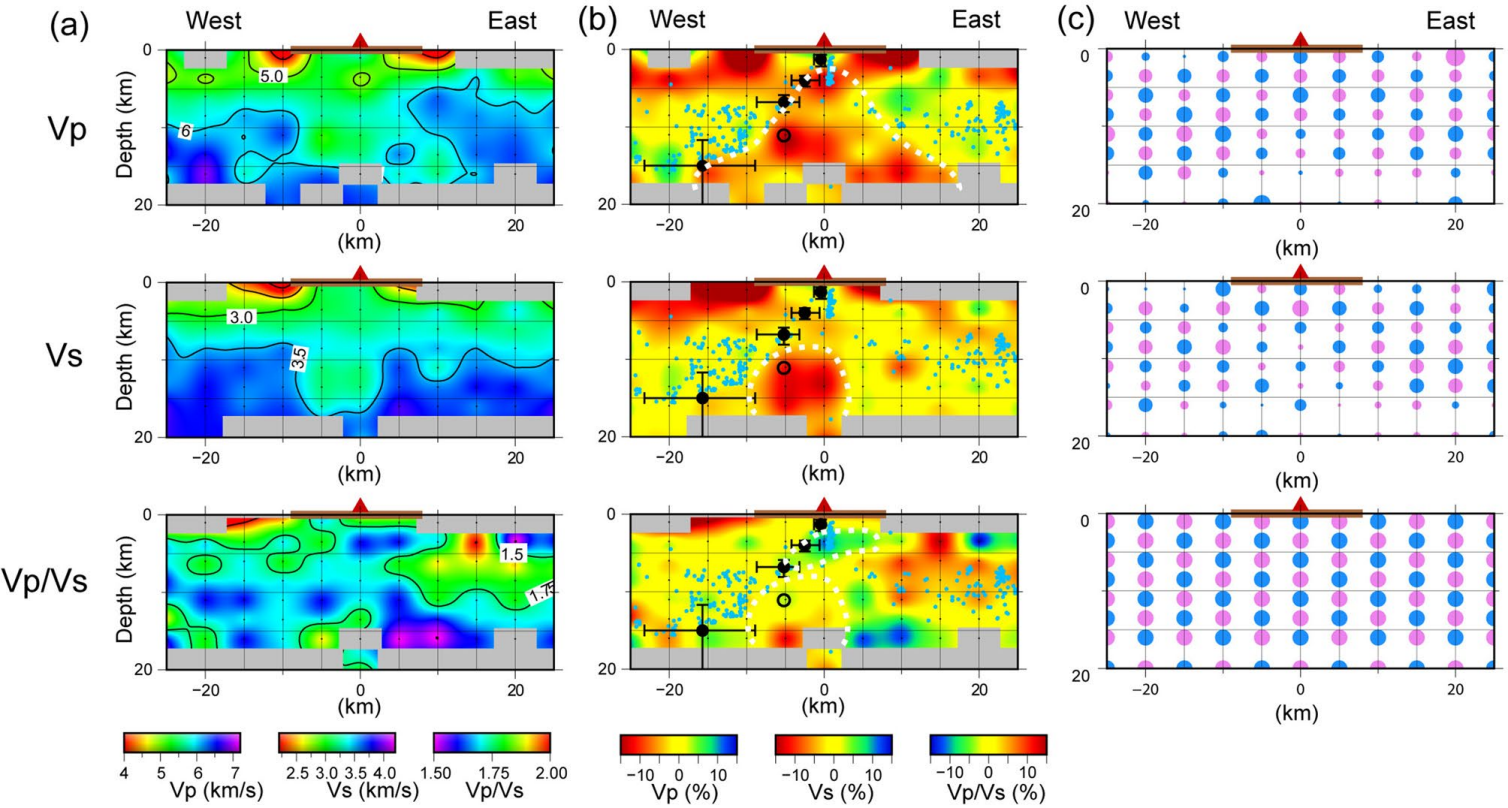
East–west cross-sections of the three-dimensional seismic velocity model across Mt. Fugendake. The red triangle and brown bar at the top of each panel represent Mt. Fugendake and Shimabara Peninsula, respectively. **(a)** Absolute values. **(b)** Perturbations from the averaged structure. The sky-blue dots indicate hypocenters used in this study that are located within 2 km of the cross-section. The low-Vp, low-Vs, low-Vp/Vs, and moderate–high Vp/Vs zones discussed in the text are shown by white dashed lines. Black solid circles with error bars and open circle indicate the pressure sources of Kohno et al. (2008) and Nishi et al. (1999), respectively. The symbols are as in Fig. 1. **(c)** Result of CRT.

We examine the attainable resolution of the tomographic inversion using a checkerboard resolution test (CRT) and a restoring resolution test (RRT). For the CRT, we constructed a model structure by assigning velocity perturbations of ± 10% to each grid node and calculate synthetic travel times for all pairs of stations and events used in the inversion. The velocity structure was then estimated from the synthetic travel times after adding random noise, within ± 0.1 s for P-wave arrivals and ± 0.2 s for S-wave arrivals, to represent picking errors. For the CRT, we employ the same damping and smoothing parameters as used for the inversion of real data. The resolution of the solution is assessed by examining whether the input checkerboard pattern of velocity perturbations is retrieved. To examine the reproduction of the checkerboard pattern quantitatively, we use a resolvability value^31^, which is the local semblance between the input and retrieved checkerboard models for a set of each grid node and its six neighboring nodes. To calculate the resolvability value, we use a 5 km operator radius from each grid. The checkerboard patterns are well recovered in areas where the resolvability value is > 0.65 (Figs. 4 and 5); accordingly, we adopt a resolvability value of 0.65 as the threshold for a well-resolved grid. Areas for which resolvability is < 0.65 are masked in Figs. 4 and 5. A comparison of CRT and DWS data (Fig. S8) indicates that areas with DWS ≥ 40 for P- and S-waves is similar to areas with resolvability values of > 0.65.

For the RRT, we calculate synthetic travel times using the final velocity structure as shown in Figs. 4 and 5. We invert these synthetic travel times again after adding the same random noise. By comparing the features of the velocity structure obtained from the observation data (i.e., the final structure as shown in Fig. 4) with those from the RRT (Fig. S9), we can confirm that the area with resolvability value of > 0.65 is robust against RRT and can therefore be considered in the following sections.

## Results and discussion

Figure 4a shows plan views of the P-wave velocity (Vp) and S-wave velocity (Vs) perturbations with respect to the DWS weighted average of the inverted velocity values at various depths. The Vp/Vs ratio is also shown as the perturbation from the initial value 1.73. Figure 4b shows plan views of the corresponding CRT results for P- and S-wave velocity structures. Figure 5 shows east–west trending vertical cross-sections of Vp, Vs, and Vp/Vs, and the results of the CRT. Below, we discuss key features of the velocity structure in and around Shimabara Peninsula. In the following, we focus on the Vp structure, which is the best constrained according to CRT. The Vs and Vp/Vs structures are used to support the interpretations based on Vp.

The horizontal slice at 1 km below sea level (b.s.l.) (Fig. 4a) shows the high-Vp and high-Vs zone west to southwest of Fugendake. In contrast, no anomalous zone is located beneath Fugendake. These results are consistent with the shallow (–1 to 2 km below sea level) Vp structure obtained from active seismic surveys around Unzen Volcano^32,33^. The high-Vp anomaly exhibits velocities between 5.0 and 5.6 km/s. One possible interpretation is that this shallow high-Vp zone reflects the lava of Younger Unzen Volcano that covers the graben structure. Drilling data show the basement of Unzen Volcano is downthrown by ~ 1 km to the west of Fugendake through displacement on normal faults within the graben (Fig. 1), which suggests that the lava sequences may be thicker beneath the westernmost portion of the volcanic edifice. However, the shallow high-Vp zone does not trend east–west (which would be expected if the structure were related to the graben) but is instead localized to the west of the volcano. Another possible interpretation is that the high-velocity core of the volcano is associated with repeated magmatic intrusions. Active seismic surveys of volcanoes worldwide have revealed that high-Vp anomalies commonly exist at a shallow level near the eruptive center^11,34–38^. These high-Vp zones have been interpreted as solidified dykes resulting from repeated intrusions throughout the history of the volcano. Similarly, we interpret the shallowest high-Vp zone of this study as a complex of solidified dykes. Because Fugendake volcano is relatively young^3^, the dyke complex may not have developed directly beneath its summit, but rather beneath the western edifice of Fugendake.

The horizontal slices at 3.5 and 6.0 km depth (Fig. 4a) show the low-Vp zone beneath the southeast, central, and northwest parts of Shimabara Peninsula. Figure 4a,c shows that the low gravity anomaly southeast of Shimabara Peninsula corresponds approximately to the low-Vp region at 3.5 km depth. Therefore, the shallow-depth low-Vp zones southeast of Shimabara Peninsula are interpreted as a zone comprising relatively low-density material. Overall, the Vs structure shows a similar pattern to the Vp structure in this depth range. However, at 3 km depth beneath the central part of Shimabara Peninsula, the low-Vp zone does not correspond to a low-Vs zone, but instead yields low Vp, moderate Vs, and low Vp/Vs. This result is inconsistent with the previous structure that shows low-Vs beneath the central part of Shimabara Peninsula at 3 km depth^22^. Note that CRT indicates that the Vs structure is well resolved at 3 km depth in this part of the peninsula. We interpret this to represent a region enriched in high-temperature volatiles. This interpretation is similar to that of Hakone Volcano^12,18^. The vertical slices (Fig. 5) show this low-Vp, moderate-Vs, and low-Vp/Vs zone is located beneath the inferred locations of the previously hypothesized shallow pressure sources.

The horizontal slices at 8.5, 11.0, and 16.0 km (Fig. 4a) indicate a low-Vp zone beneath Fugendake. The low-Vp zone extends east and west from Shimabara Peninsula as the low-Vp zone becomes deeper (Figs. 4a and 5a). These deep features are generally consistent with the results of previous studies^22,23^. It is also consistent with the previous study^22^ that the low-Vp zone exists at depth to the southwest of Shimabara Peninsula. Of note, most of the tectonic earthquakes occur above the deep low-Vp zone, as did the migration of hypocenters prior to the eruptions in 1990–1995. This spatial relationship suggests that the deep low-Vp zone corresponds to a zone of high temperature (> 400 °C) above the brittle–ductile transition^39^. Another important observation is that the deepest pressure sources are located at the top of the deep low-Vp zone. From this observation we infer that magma associated with the 1990–1995 eruptions migrated along the upper boundary of the low-Vp high-temperature zone. Since the shallow pressure sources are also located at the top of the shallow low-Vp zone, we conclude that the magma of the 1990–1995 eruptions also migrated along the top of the low-Vp zone beneath Fugendake. We also find that the low-Vs zone is present at depths of 8–12 km beneath Fugendake, despite no low-Vs anomalies being reported in previous work^22^.

The relationship between the low-Vp zone imaged in this study and the locations of tectonic earthquakes and previously modeled pressure sources is also apparent in the high-resolution seismic structure of Hakone volcano, Japan^12,18^. From the Vp, Vs, and Vp/Vs structure, Yukutake et al. (2015, 2021) interpreted the shallower part of a low-Vp anomaly to be a fluid- or gas-rich zone (characterized by low Vp, low Vs, and low Vp/Vs), while the deeper part of the anomaly (characterized by low Vp, low Vs, and high Vp/Vs) was inferred to be a zone of highly crystallized magma with a melt content of ~ 4%. The low-Vp zone beneath Fugendake similarly shows low Vp/Vs at shallow levels (3–7 km depth) and moderate to high Vp/Vs at deeper levels (7–16 km; Fig. 5b). Accordingly, at Fugendake we infer a shallow, volatile-rich zone overlying a zone of deeper, highly crystalized magma. The pressure source of Hakone Volcano is thought to be volatiles trapped beneath a silica sealing layer at the brittle–ductile transition^18^. According to this interpretation, the magma of the 1990–1995 eruptions at Unzen may have migrated obliquely upward along the bottom of a similar silica sealing layer, although further supporting evidence is necessary to fully understand the oblique ascent of magma beneath Fugendake.

Our results are inconclusive as to the provenance of the magma. The results of the CRT show that the structure around Tachibana Bay, where a magma reservoir might exist, is well resolved (Fig. 4b). Previous reports of crustal deformation^6^ and earthquake migration^4^ during 1989–1991, associated with the onset of eruptive activity at Unzen, suggest that the primary magma reservoir is located beneath Tachibana Bay. However, our tomography data to a depth of 16 km beneath Tachibana Bay do not show a low-Vp, low-Vs, and high-Vp/Vs region indicative of a melt-rich zone^10,12^. The primary magma reservoir might be too small to be resolved by seismic tomography or might be located at depths greater than 16 km. Another possible explanation is that the primary magma is not located beneath Tachibana Bay, but instead occurs beneath Fugendake, corresponding to the large low-Vp anomaly identified in this study. To better resolve the location of the primary reservoir and to fully understand the oblique magma migration route along the top of low-Vp zone, we suggest that new observation sites are required to improve the resolution of the Vp and Vs structure. New three-component sites around the northern and southern coastlines of Shimabara Peninsula and on the small island to the southeast would improve the S-wave resolution, especially at depths greater than 10 km. New magnetotelluric observations that supplement the electrical resistivity data of previous studies^40–42^ and the application of intrinsic and scattering seismic attenuation methods^43^ to Unzen volcano would also help to image the 3D subsurface and interpret the low-Vp zone beneath Shimabara Peninsula described in this study.

## Conclusions

We used high-resolution seismic tomography to image a low-Vp zone beneath Unzen Volcano, Japan, at depths of 3–16 km. The majority of recorded tectonic earthquakes occur above the deeper portion (7–16 km) of this low-Vp anomaly; accordingly, we interpret this zone as a high-temperature (> 400 °C) region overlying the brittle–ductile transition. The shallower part of this anomaly (3–7 km) is characterized by low Vp, low Vs, and low Vp/Vs. Previously reported pressure source locations coincide with the uppermost parts of both the deep and shallow low-Vp zones, which leads us to conclude that the magma associated with the 1990–1995 eruptions migrated obliquely upward along the top of the low-Vp anomaly. We did not retrieve a low-Vp, low-Vs, and high-Vp/Vs region beneath Tachibana Bay, where previous studies have proposed the existence of the primary magma reservoir.

## Supplementary Information

Supplementary Figures.
