# Supplementary material for: Seismic velocity structure of Unzen Volcano, Japan, and relationship to the magma ascent route during eruptions in 1990–1995
Source: Sci Rep. 2021 Nov 17;11:22407. doi: 10.1038/s41598-021-00481-6 (PMC8599693; doi:10.1038/s41598-021-00481-6)
Supplement: Supplementary file 1 — Supplementary Figures. [file 41598_2021_481_MOESM1_ESM.pdf]

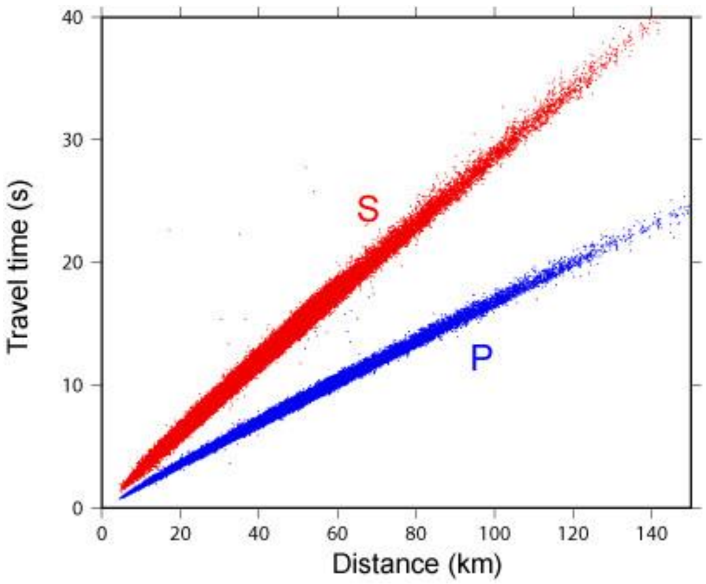

Figure S1. P-wave and S-wave travel time respect to the distance. Horizontal axis shows the distance between a hypocenter and an observation site.

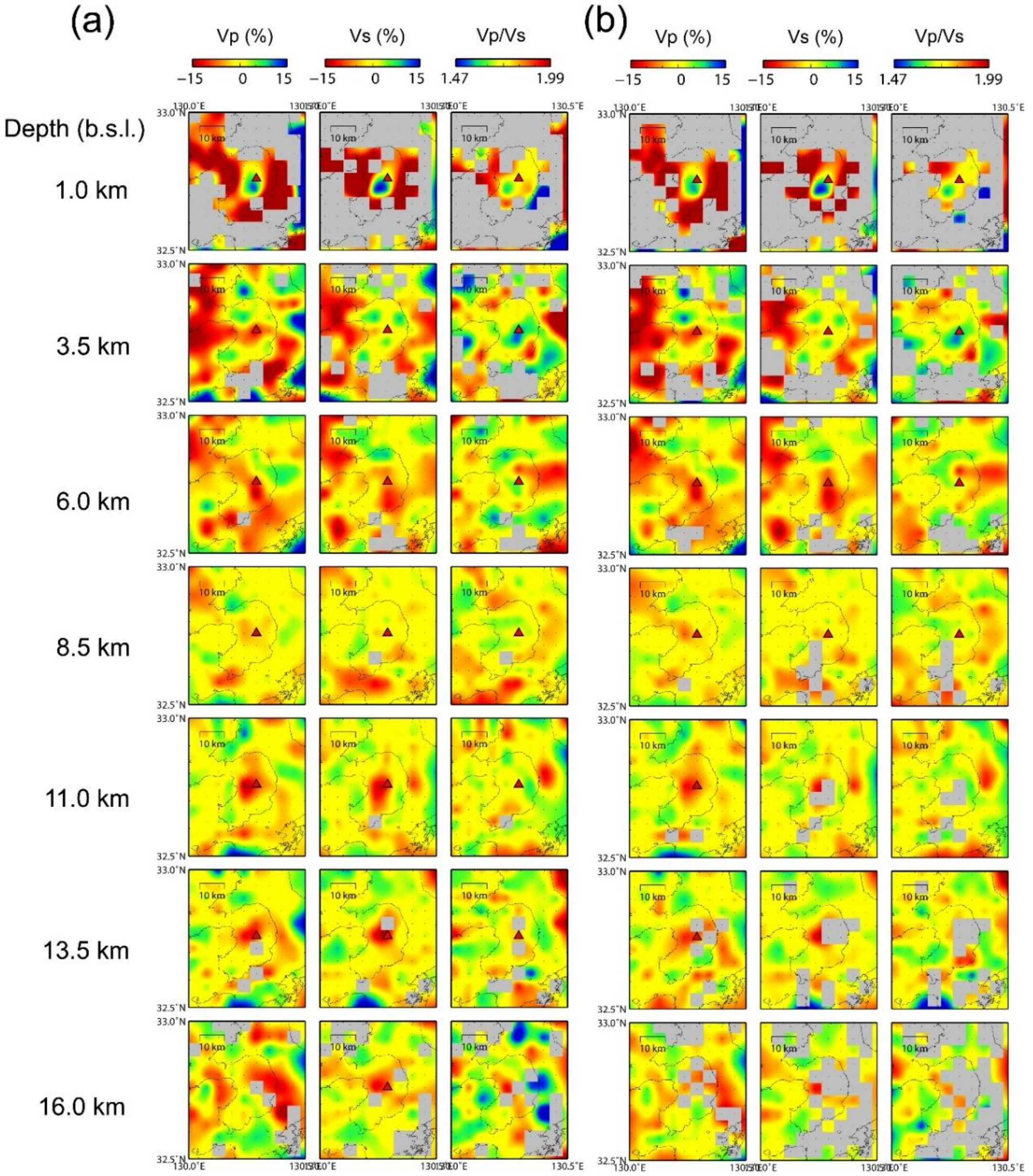

Figure S2. Horizontal slices of P-wave, S-wave, and Vp/Vs perturbations at various depths (beneath sea level; b.s.l.). Regions of lower reliability with resolvability values of <0.65 are masked (gray). (a) Seismic velocity structure, as in Fig. 4. (b) Structures from single-step inversion using the travel time difference from pairs of earthquakes within 3 km of each other.

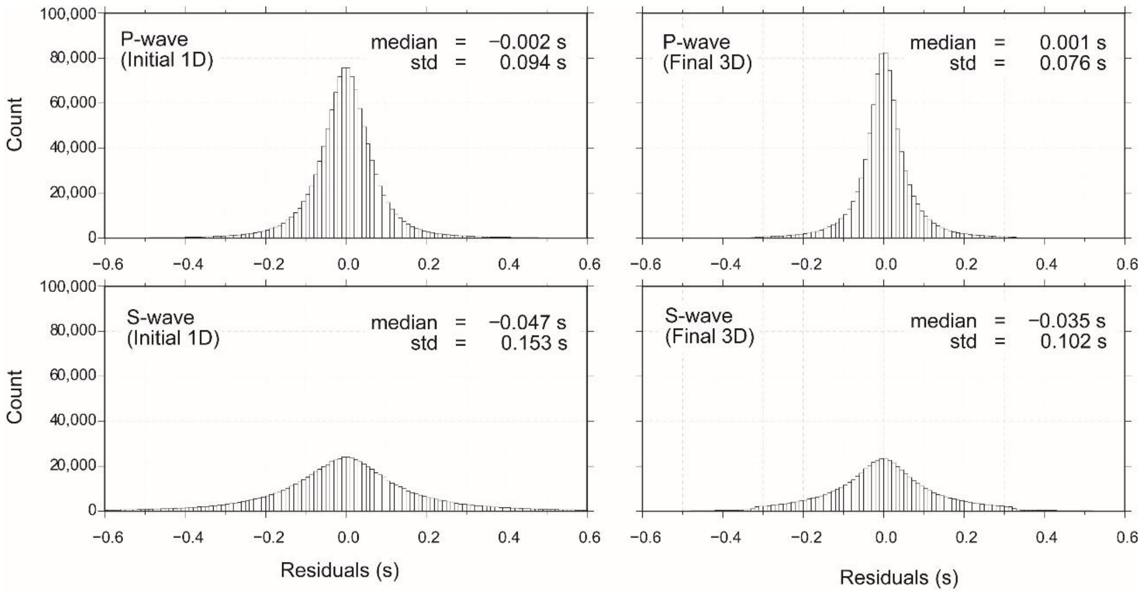

Figure S3. Histograms for P-wave and S-wave travel-time residual. Summary statistics (median and standard deviation) are provided in the upper right.

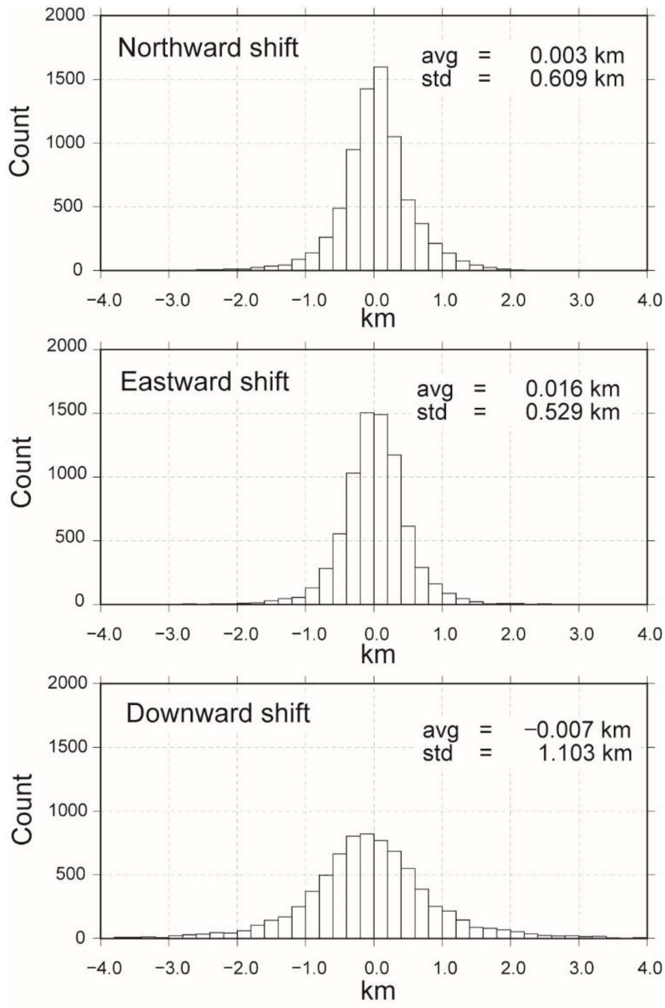

Figure S4. Histograms for the shift of the hypocenters from the initial one to the relocated one. Summary statistics (average and standard deviation) are provided in the upper right.

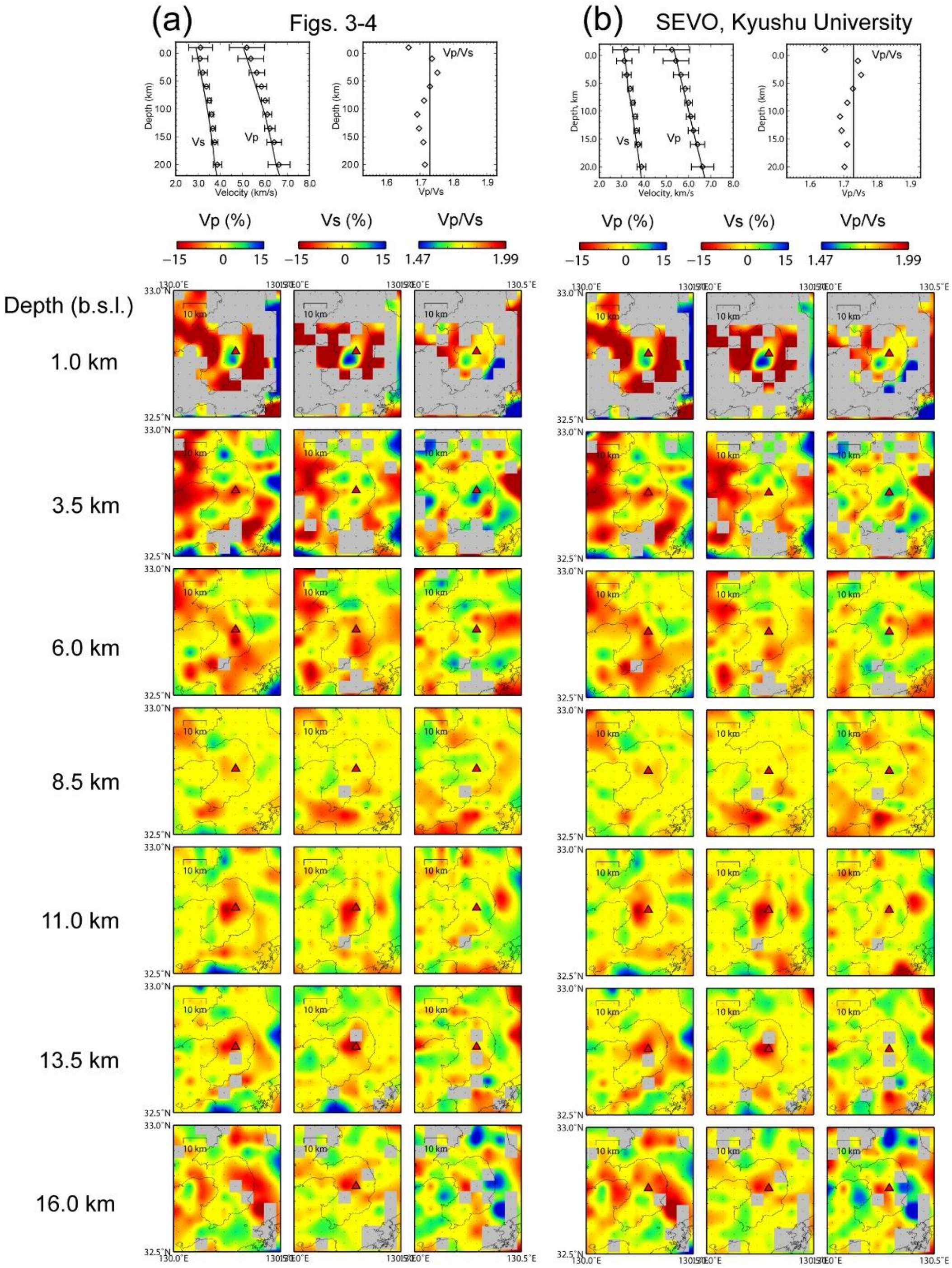

Figure S5. Comparison of the final seismic velocity structure and the one that use different initial structure in the inversion. (a) Seismic velocity structure, as in Fig.3 and Fig. 4a. (b) Seismic velocity structure by using the initial 1-D structure from Kyushu University. No significant difference is seen in the region of high reliability (unmasked area).

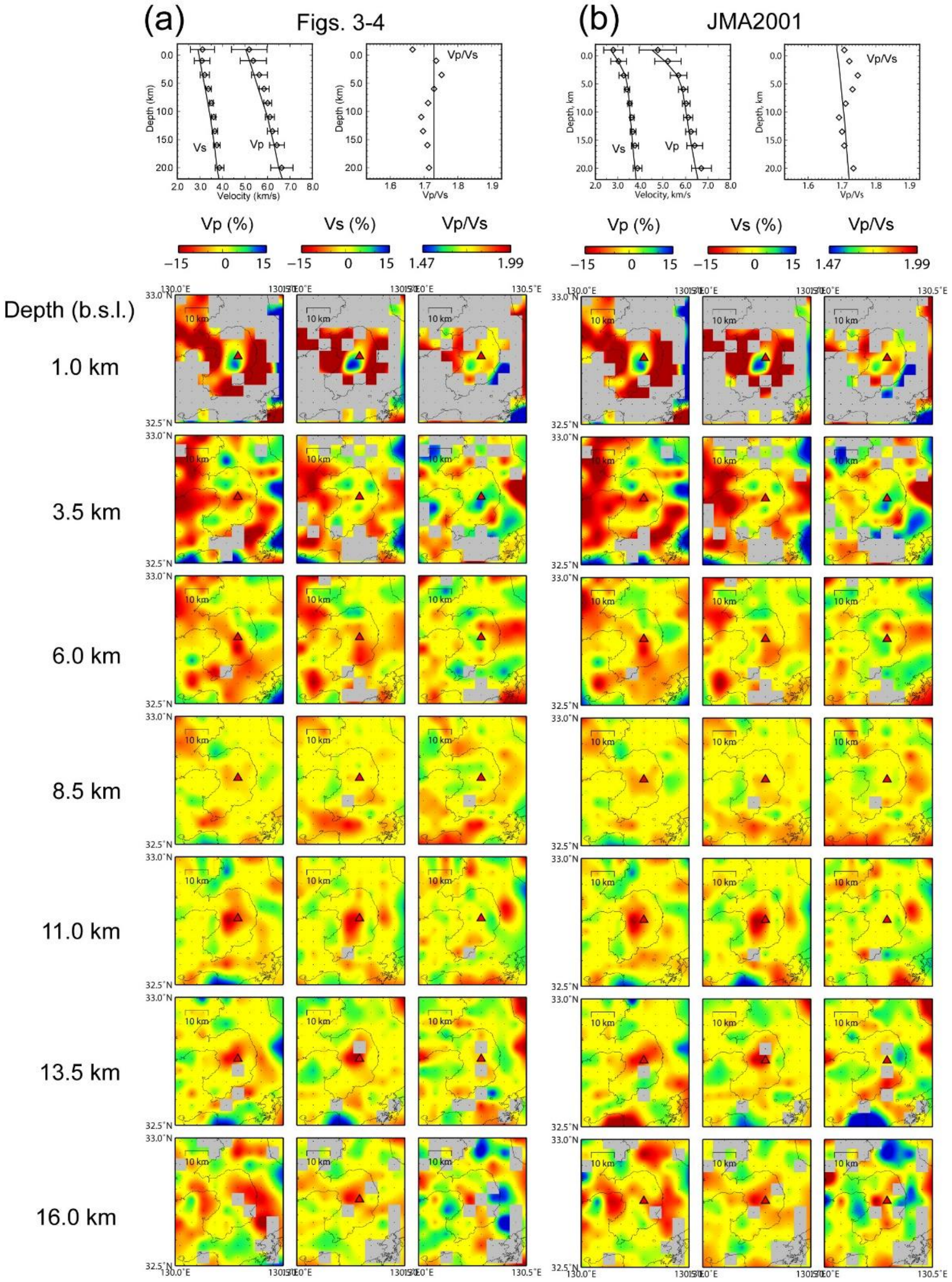

Figure S6. Comparison of the final seismic velocity structure and the one that use different initial structure in the inversion. (a) Seismic velocity structure, as in Fig.3 and Fig. 4a. (b) Seismic velocity structure by using the initial 1-D structure (JMA2001) from Japan Meteorological Agency (JMA). No significant difference is seen in the region of high reliability (unmasked area).

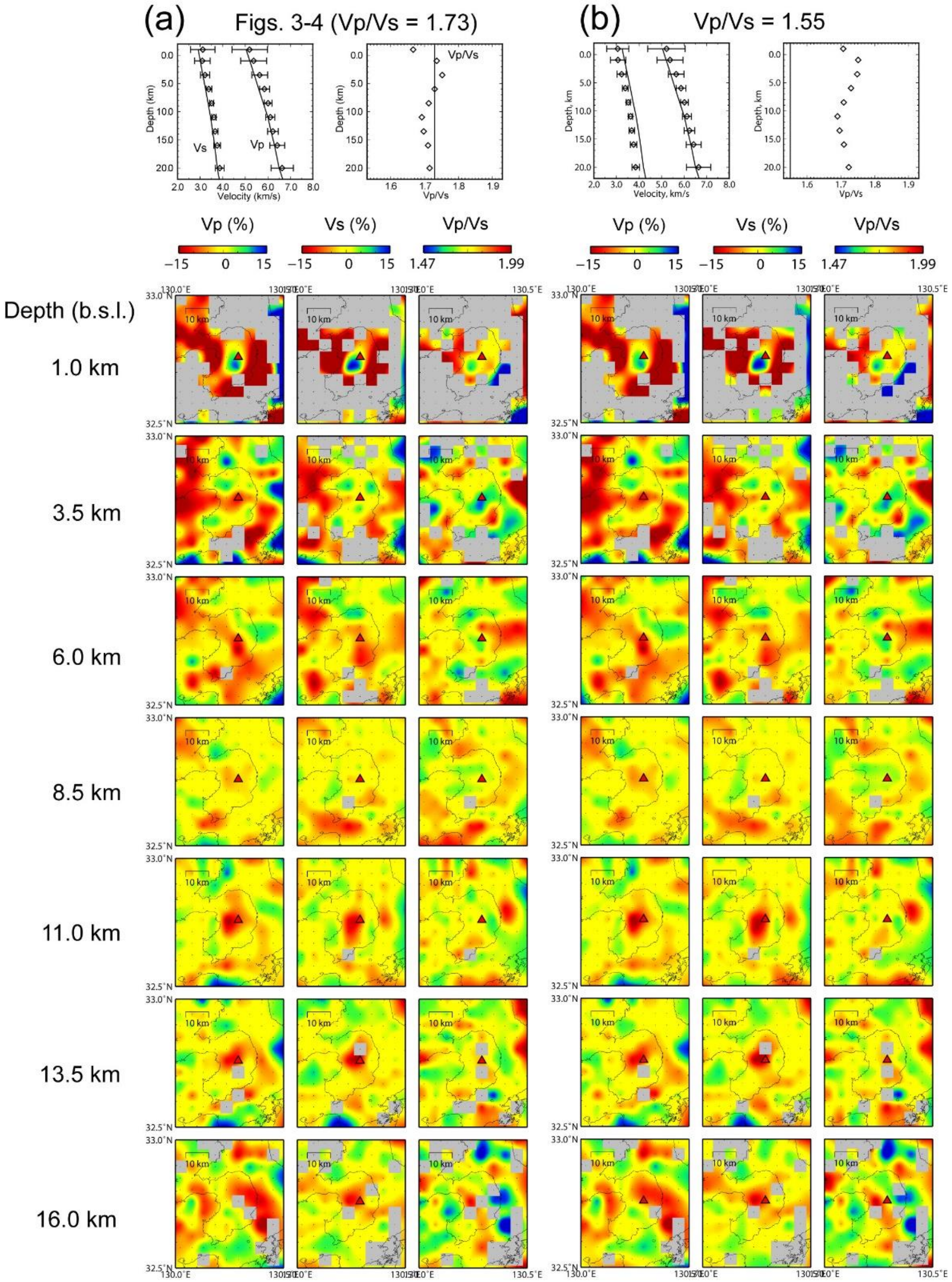

Figure S7. Comparison of the final seismic velocity structure and the one that use different initial structure in the inversion. (a) Seismic velocity structure, which use initial Vp/Vs of 1.73, as in Fig.3 and Fig. 4a. (b) Seismic velocity structure by using the initial Vp/Vs of 1.55. No significant difference is seen in the region of high reliability (unmasked area).

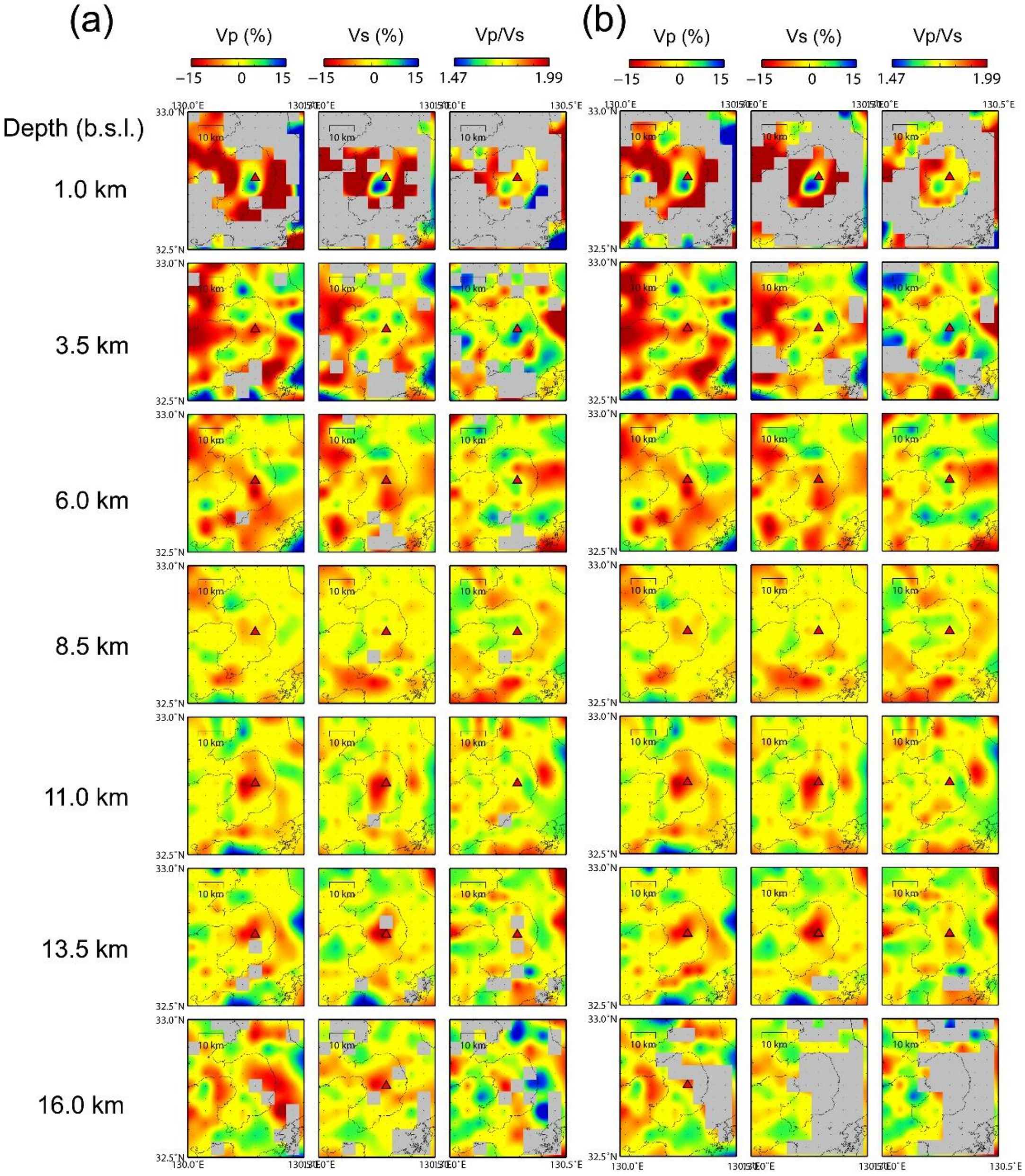

Figure S8. Horizontal slices of P-wave, S-wave, and Vp/Vs perturbations at various depths (beneath sea level; b.s.l.). (a) Areas with resolvability value < 0.65 are masked. (b) Areas with Derivative Weighted Sum (DWS) < 43 are masked.

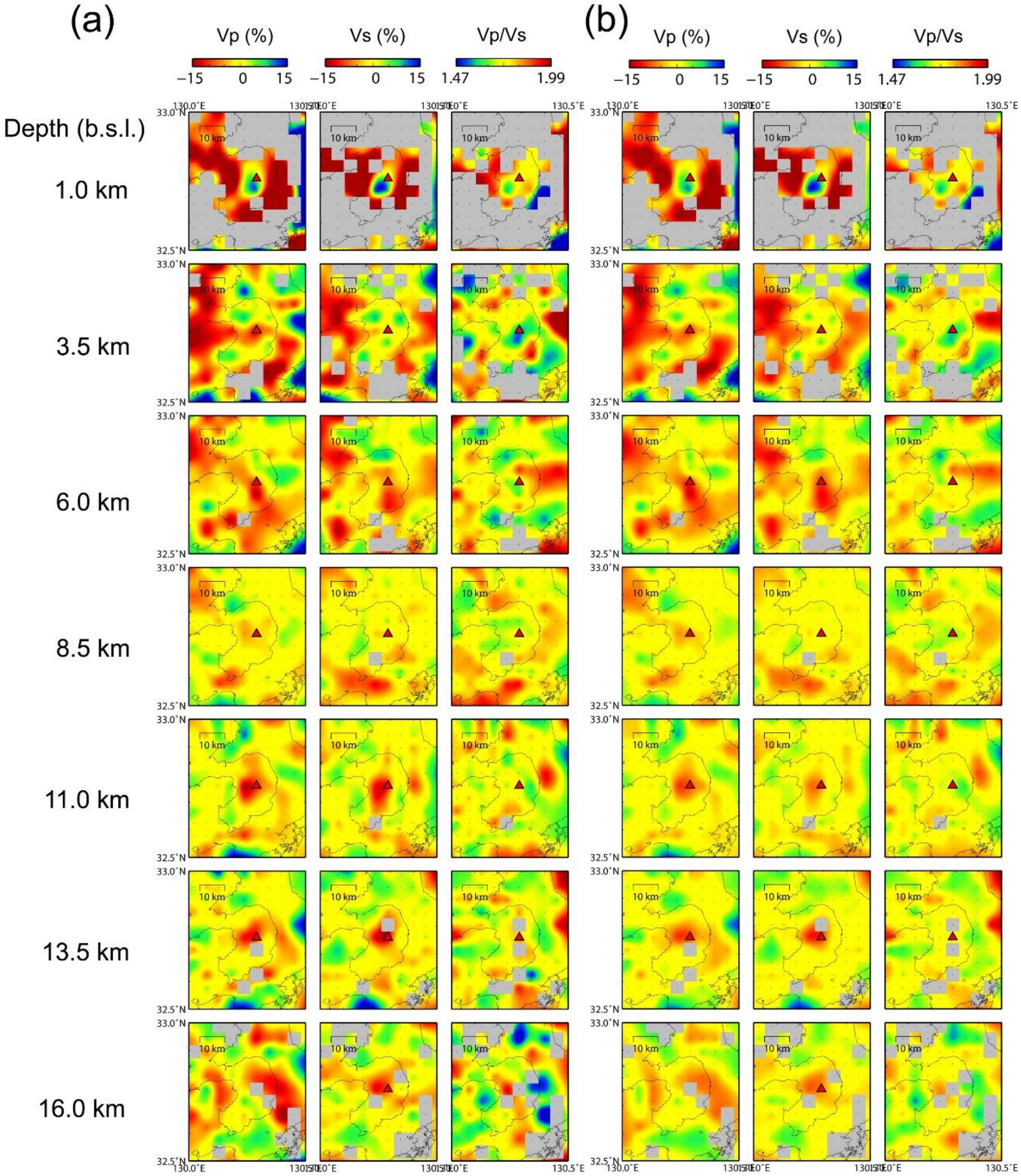

Figure S9. Results of restoring resolution test (RRT). (a) Horizontal slices of P-wave, S-wave, and Vp/Vs perturbations at various depths (beneath sea level; b.s.l.). The seismic velocity structure is as in Fig. 4. (b) Result of the RRT.
